# What is the true incidence of renal artery stenosis after sympathetic denervation?

**DOI:** 10.3389/fphys.2014.00311

**Published:** 2014-08-15

**Authors:** Yutang Wang

**Affiliations:** ^1^Queensland Research Centre for Peripheral Vascular Disease, School of Medicine and Dentistry, James Cook UniversityTownsville, QLD, Australia; ^2^School of Health Sciences, Federation University AustraliaMount Helen, VIC, Australia

**Keywords:** clinical trials, renal artery stenosis, renal denervation, resistant hypertension, side effect

Renal denervation (RDN), a recently developed therapy for resistant hypertension, is generally regarded as a safe procedure (Krum et al., 2009; Esler et al., 2010; Bhatt et al., 2014). The Symplicity HTN trials reported that the rate of renal artery stenosis after RDN was low. For example, the Symplicity HTN-1 trial showed that 1 of 45 (2.2%) denervated patients developed a non-obstructive renal artery stenosis in an untreated area at 6 months after RDN (Krum et al., 2009). This low rate of renal artery stenosis after RDN was confirmed by the 6-month report of the Symplicity HTN-2 trial (*N* = 106) (Esler et al., 2010) and the recently published 6-month report of the Symplicity HTN-3 trial (*N* = 535) (Bhatt et al., 2014), which was 1.9 and 0.3%, respectively. In these Symplicity HTN trials, renal artery stenosis occurred at a low rate (0.3–2.2%) and was not reported to cause further complications.

However, some studies reported that renal artery stenosis occurred at a higher rate. For example, the EnligHTN I study (Worthley et al., 2013) reported that 2 of 46 (4.3%) patients showed progression of a pre-existing renal artery stenosis; a study from 10 European expert centers (Persu et al., 2014) reported that 3 of 109 (2.8%) patients showed progression of a non-significant (<30%) renal artery stenosis; and Versaci et al. (2014a) reported that 2 of 11 (18.2%) patients developed severe renal artery stenosis. It is worthwhile to point out that the sample size of 11 in Versaci et al.'s report is relatively small (Versaci et al., 2014a).

In 2012, Vonend et al. reported that a patient developed a 75% stenosis near the ostium of the right renal artery, which caused recurrent hypertension (Vonend et al., 2012). Subsequently, another 3 case reports reported that renal artery stenosis after RDN caused recurrent hypertension (Kaltenbach et al., 2012; Aguila et al., 2014; Pucci et al., 2014).

The causal role of RDN in promoting renal artery stenosis is currently speculative. However, given that (1) renal artery stenosis causes recurrent hypertension in denervated patients (Kaltenbach et al., 2012; Vonend et al., 2012; Aguila et al., 2014; Pucci et al., 2014) and (2) treatment of renal artery stenosis is not always safe and sometimes leads to death (Soriano-Perez et al., 2012), it is important to thoroughly investigate the effect of RDN on renal artery stenosis (Mahfoud and Kjeldsen, 2013; Wang, 2014a,b,c,d). To do this, the following three points need to be emphasized in future clinical trials on RDN:

1. Long-term randomized trials are needed. Two major randomized trials on RDN, i.e., the Symplicity HTN-2 and HTN-3 trials (Esler et al., 2012; Kandzari et al., 2012), allowed patients in the randomized control group to receive RDN after completion of the 6-month study. This crossover design makes it difficult to investigate possible long-term side effects of RDN, e.g., promoting renal artery stenosis. Therefore, long-term randomized trials without a short-term crossover design are needed to investigate the effect of RDN on renal artery stenosis.
2. Imaging methods monitoring renal artery stenosis need to be standardized. Symplicity HTN trials did not standardize renal artery imaging methods during follow ups. Ultrasonography, magnetic resonance angiography, and computerized tomographic angiography were used (Krum et al., 2009; Esler et al., 2010). The computerized tomographic angiography, the gold standard method to detect renal artery stenosis (Persu et al., 2012), was not the major imaging method in these trials. It is known that ultrasonography has limited visualization on renal artery stenosis because (1) the imaging is interfered by overlying adipose tissue and bowel gas (Zhang et al., 2009); and (2) the entire length of the renal artery or an accessory renal artery can be overlooked (Lao et al., 2011). Therefore, it is worthwhile to use computerized tomographic angiography as the standardized method during follow ups in future trials to investigate the effect of RDN on renal artery stenosis.
3. It is likely that improved catheters for RDN using lower power radiofrequency over a shorter time will reduce the local tissue injury at the ablation site compared with that caused by the first generation RDN systems (Versaci et al., 2014b). However data regarding the vascular injury induced by these new devices are lacking.

## Conflict of interest statement

The author declares that the research was conducted in the absence of any commercial or financial relationships that could be construed as a potential conflict of interest.
